# Neutrophils, Fast and Strong 2.0: Heterogeneity of Neutrophil Parameters in Health and in Disease

**DOI:** 10.3390/biomedicines13020436

**Published:** 2025-02-11

**Authors:** Galina F. Sud’ina

**Affiliations:** Belozersky Institute of Physico-Chemical Biology, Lomonosov Moscow State University, Moscow 119234, Russia; sudina@genebee.msu.ru

## 1. Introduction

Neutrophils are very important cells of the immune system, and every year, new nuances of their functional activity in the body and participation in various pathological processes are discovered. Detailed characterization of neutrophils and other immune cells can help identify pathologies at early stages. Elevated neutrophil levels often correlate with pathologies. Absolute neutrophil counts are increased in obesity [1], in patients with bacteremia [2], in a non-survival group in patients with colorectal cancer [3], and are associated with poor cognitive functions in older adults [4]. At the same time, a higher neutrophil count is associated with a lower risk of venous thromboembolism [5].

One of the most important characteristics and markers of health is the proportion of neutrophils in relation to other cells involved in the immune response. The level of neutrophils in relation to other immune cells can provide a more accurate characterization of the body since it allows one to identify an imbalance of immunity during inflammatory processes. Elevated neutrophil-to-lymphocyte ratio (NLR) is a prognostic marker of mortality in diabetes [6]. NLR may predict thrombosis risk [7].

Currently, the heterogeneity of neutrophils is widely recognized, and numerous studies have been devoted to this topic. Increased neutrophil size was observed in patients with sepsis and trauma [8]. Mean neutrophil volume is altered in sepsis when immature neutrophils enter the circulation [9]. Tumor-associated neutrophils are heterogeneous [10], and neutrophils from patients who have undergone lung transplantation are heterogeneous [11]. Recently, it was elegantly demonstrated how the spleen supports neutrophil heterogeneity during emergency granulopoiesis [12].

The papers presented in this Special Issue examine various aspects of neutrophil heterogeneity, including differences in their balance with lymphocytes and platelets.

## 2. An Overview of Published Articles

Patients with liver disease have a high need for hemodialysis. For successful hemodialysis, the arteriovenous fistula (AVF) must operate at a certain flow rate [13]. In the work of Edoardo Pasqui et al. (contribution 1), neutrophil-to-lymphocyte ratios (NLR) and platelet-to-lymphocyte ratios (PLR) were investigated as biomarkers, and high NLR and PLR values were found to be significant risk factors for AVF failure.

In diabetic ketoacidosis (DKA), high NLR levels lead to severe cerebral edema (Alexandra-Cristina Scutca, et al., contribution 3). The main causes of DKA are neurological [14]. However, complications may also be associated with tissue destruction by enzymes from neutrophilic azurophilic granules [15]. In an earlier publication, Alexander-Christian Scutz et al. first showed that high NLR levels are a very informative marker of DKA [16].

When various neutrophil parameters were examined, neutrophil size was lowest in the healthy group (Elżbieta Rutkowska et al., contribution 4). Complexity of the neutrophil population has been observed in patients with lung cancer (contribution 4). Indicator of inflammation neutrophil reactivity index, which is known to be increased in sepsis [17], was decreased in COVID-19 (contribution 4).

Neutrophils are highly heterogeneous across tumor types. Cancer progression depends on the neutrophil activation profile [18]. Neutrophils respond very quickly to activation, and it is very important to have some checkpoints to assess their activation status. Khetam Sunbuli et al. (contribution 7) conducted a detailed study and identified Tbp and Hprt1 as the respective reference genes for mouse splenic neutrophils regardless of their activation status.

Neutrophils circulate in our bodies in a naturally inactive state until they receive a signal from an activated endothelium in the body’s vessels [19,20]. This is followed by the migration of neutrophils to the site of inflammation. The inflammatory state often correlates with levels of inflammatory biomarkers such as C-reactive protein [21]. In severe pathologies, including pneumonia, the interaction of neutrophils with the endothelium leads to endothelial damage and other severe complications [22]. Paula Gonzalez-Jimenez et al. (contribution 5) classified biomarkers of endothelial injury and compared them with traditional inflammatory biomarkers. For the first time (contribution 5), the biomarker mid-regional proadrenomedullin [MR-proADM] was shown to be the best predictor of complications in pneumonia.

In the fight against bacteria, neutrophils release DNA strands called neutrophil extracellular traps (NETs) [23] and also use secretory outgrowths containing bactericides-cytonemes [24]. NETs are released by dying neutrophils. The neutrophil cell death pathway through NETosis causes complications in many diseases, including diabetic foot ulcer pathology [25] and vascular complications during infection [26], and leads to immunothrombosis in COVID-19 [27]. Yuichi Watanabe et al. (contribution 6) found that oxysterol suppressed DNA release from neutrophils.

Neutrophils produce reactive oxygen species (ROS) at stimulation and in interaction with bacteria. Given the bactericidal activity of neutrophils, it is very important to know the production of ROS by individual cells. In the work of Svetlana N. Pleskova et al. (contribution 2), a method for detecting reactive oxygen species has been created at the single-cell level. When studying the interaction of neutrophils with *S. aureus* and *E. coli* bacteria, the authors found functional heterogeneity in the neutrophil population: the peak response time differs between individual cells and the neutrophil population.

## 3. Conclusions

The articles in this Special Issue make a valuable contribution to the study of neutrophil parameters in health and various pathologies.

## References

[1] Gomez-Casado G., Jimenez-Gonzalez A., Rodriguez-Munoz A., Tinahones F.J., Gonzalez-Mesa E., Murri M., Ortega-Gomez A. (2024). Neutrophils as indicators of obesity-associated inflammation: A systematic review and meta-analysis. Obes. Rev..

[2] Sullivan E., Schulte R., Speaker S.L., Sabharwal P., Wang M., Rothberg M.B. (2024). Relationship Between White Blood Cell Count and Bacteremia Using Interval Likelihood Ratios in Hospitalized Patients. J. Gen. Intern. Med..

[3] Garcia-Flores L.A., De Vera M.T.D., Pilo J., Rego A., Gomez-Casado G., Arranz-Salas I., Martín I.H., Alcaide J., Torres E., Ortega-Gomez A. (2024). Increased neutrophil counts are associated with poor overall survival in patients with colorectal cancer: A five-year retrospective analysis. Front. Immunol..

[4] Fa W., Liang X., Liu K., Wang N., Liu C., Tian N., Zhu M., Ma Y., Song L., Tang S. (2024). Associations of Blood Absolute Neutrophil Count and Cytokines with Cognitive Function in Dementia-Free Participants: A Population-Based Cohort Study. J. Gerontol. Ser. A.

[5] Zhao J., Zhang W., Wei H., Liu S., Cui S., Chen S. (2024). Neutrophil count and reduced risk of venous thromboembolism: A Mendelian randomization study. Hematology.

[6] Dabaghi G.G., Rad M.R., Mortaheb M., Darouei B., Amani-Beni R., Mazaheri-Tehrani S., Izadan M., Touhidi A. (2024). The Neutrophil-to-Lymphocyte Ratio Predicts Cardiovascular Outcomes in Patients with Diabetes: A Systematic Review and Meta-Analysis. Cardiol. Rev..

[7] Ripamonti A., Cavalca F., Montelisciani L., Antolini L., Gambacorti-Passerini C., Elli E.M. (2024). Neutrophil-to-lymphocyte ratio (NLR) at diagnosis in essential thrombocythemia: A new promising predictor of thrombotic events. Cancer.

[8] Joosse H.J., Huisman A., van Solinge W., Hietbrink F., Hoefer I., Haitjema S. (2022). Describing Characteristics and Differences of Neutrophils in Sepsis, Trauma, and Control Patients in Routinely Measured Hematology Data. Biomedicines.

[9] Mishra A., Jena P.K., Panda S.K. (2024). The diagnostic performance of mean neutrophil volume in neonatal sepsis: A systematic review and meta-analysis. Pediatr. Neonatol..

[10] Teo J.M.N., Chen Z., Chen W., Tan R.J.Y., Cao Q., Chu Y., Ma D., Chen L., Yu H., Lam K.H. (2025). Tumor-associated neutrophils attenuate the immunosensitivity of hepatocellular carcinoma. J. Exp. Med..

[11] Cambier S., Beretta F., Nooyens A., Metzemaekers M., Portner N., Kaes J., de Carvalho A.C., Cortesi E.E., Beeckmans H., Hooft C. (2024). Heterogeneous neutrophils in lung transplantation and proteolytic CXCL8 activation in COVID-19, influenza and lung transplant patient lungs. Cell. Mol. Life Sci..

[12] Guo R., Xie X., Ren Q., Liew P.X. (2024). New insights on extramedullary granulopoiesis and neutrophil heterogeneity in the spleen and its importance in disease. J. Leukoc. Biol..

[13] Sidawy A.N., Gray R., Besarab A., Henry M., Ascher E., Silva M., Miller A., Scher L., Trerotola S., Gregory R.T. (2002). Recommended standards for reports dealing with arteriovenous hemodialysis accesses. J. Vasc. Surg..

[14] Jeziorny K., Waszczykowska A., Baranska D., Szadkowska A., Mlynarski W., Zmyslowska A. (2020). Can we effectively predict the occurrence of cerebral edema in children with ketoacidosis in the course of type 1 diabetes?—Case report and literature review. J. Pediatr. Endocrinol. Metab..

[15] Woo M.M., Patterson E.K., Clarson C., Cepinskas G., Bani-Yaghoub M., Stanimirovic D.B., Fraser D.D. (2016). Elevated Leukocyte Azurophilic Enzymes in Human Diabetic Ketoacidosis Plasma Degrade Cerebrovascular Endothelial Junctional Proteins. Crit. Care Med..

[16] Scutca A.C., Nicoara D.M., Marazan M., Brad G.F., Marginean O. (2022). Neutrophil-to-Lymphocyte Ratio Adds Valuable Information Regarding the Presence of DKA in Children with New-Onset T1DM. J. Clin. Med..

[17] Lee J., Gu J., Seo J.E., Kim J.W., Kim H.K. (2023). Diagnostic and Prognostic Values of Neutrophil Reactivity Intensity (NEUT-RI) in Pediatric Systemic Inflammatory Response Syndrome and Sepsis. Ann. Clin. Lab. Sci..

[18] Ekstedt S., Piersiala K., Kolev A., Farrajota Neves da Silva P., Margolin G., Kumlien Georen S., Cardell L.O. (2024). Phenotypical differences of neutrophils patrolling tumour-draining lymph nodes in head and neck cancer. Br. J. Cancer.

[19] Marki A., Esko J.D., Pries A.R., Ley K. (2015). Role of the endothelial surface layer in neutrophil recruitment. J. Leukoc. Biol..

[20] Filippi M.D. (2019). Neutrophil transendothelial migration: Updates and new perspectives. Blood.

[21] Stef A., Bodolea C., Bocsan I.C., Cainap S.S., Achim A., Serban A., Solomonean A.G., Tintiuc N., Buzoianu A.D. (2024). The Value of Biomarkers in Major Cardiovascular Surgery Necessitating Cardiopulmonary Bypass. Rev. Cardiovasc. Med..

[22] Liu D., Langston J.C., Prabhakarpandian B., Kiani M.F., Kilpatrick L.E. (2023). The critical role of neutrophil-endothelial cell interactions in sepsis: New synergistic approaches employing organ-on-chip, omics, immune cell phenotyping and in silico modeling to identify new therapeutics. Front. Cell. Infect. Microbiol..

[23] Brinkmann V., Reichard U., Goosmann C., Fauler B., Uhlemann Y., Weiss D.S., Weinrauch Y., Zychlinsky A. (2004). Neutrophil extracellular traps kill bacteria. Science.

[24] Galkina S.I., Fedorova N.V., Golenkina E.A., Stadnichuk V.I., Sud’ina G.F. (2020). Cytonemes Versus Neutrophil Extracellular Traps in the Fight of Neutrophils with Microbes. Int. J. Mol. Sci..

[25] Harithpriya K., Kaussikaa S., Kavyashree S., Geetha A., Ramkumar K.M. (2024). Pathological insights into cell death pathways in diabetic wound healing. Pathol. Res. Pract..

[26] Kolaczkowska E., Jenne C.N., Surewaard B.G., Thanabalasuriar A., Lee W.Y., Sanz M.J., Mowen K., Opdenakker G., Kubes P. (2015). Molecular mechanisms of NET formation and degradation revealed by intravital imaging in the liver vasculature. Nat. Commun..

[27] Middleton E.A., He X.Y., Denorme F., Campbell R.A., Ng D., Salvatore S.P., Mostyka M., Baxter-Stoltzfus A., Borczuk A.C., Loda M. (2020). Neutrophil extracellular traps contribute to immunothrombosis in COVID-19 acute respiratory distress syndrome. Blood.

